# Supplementary material for: The Construction and Exploration of a Comprehensive MicroRNA Centered Regulatory Network in Foxtail Millet (Setaria italica L.)
Source: Front Plant Sci. 2022 May 6;13:848474. doi: 10.3389/fpls.2022.848474 (PMC9121102; doi:10.3389/fpls.2022.848474)
Supplement: Supplementary file 1 [file Data_Sheet_1.zip › Sit-miRN3027_Seita.5G456500.1_2388_TPlot.pdf]

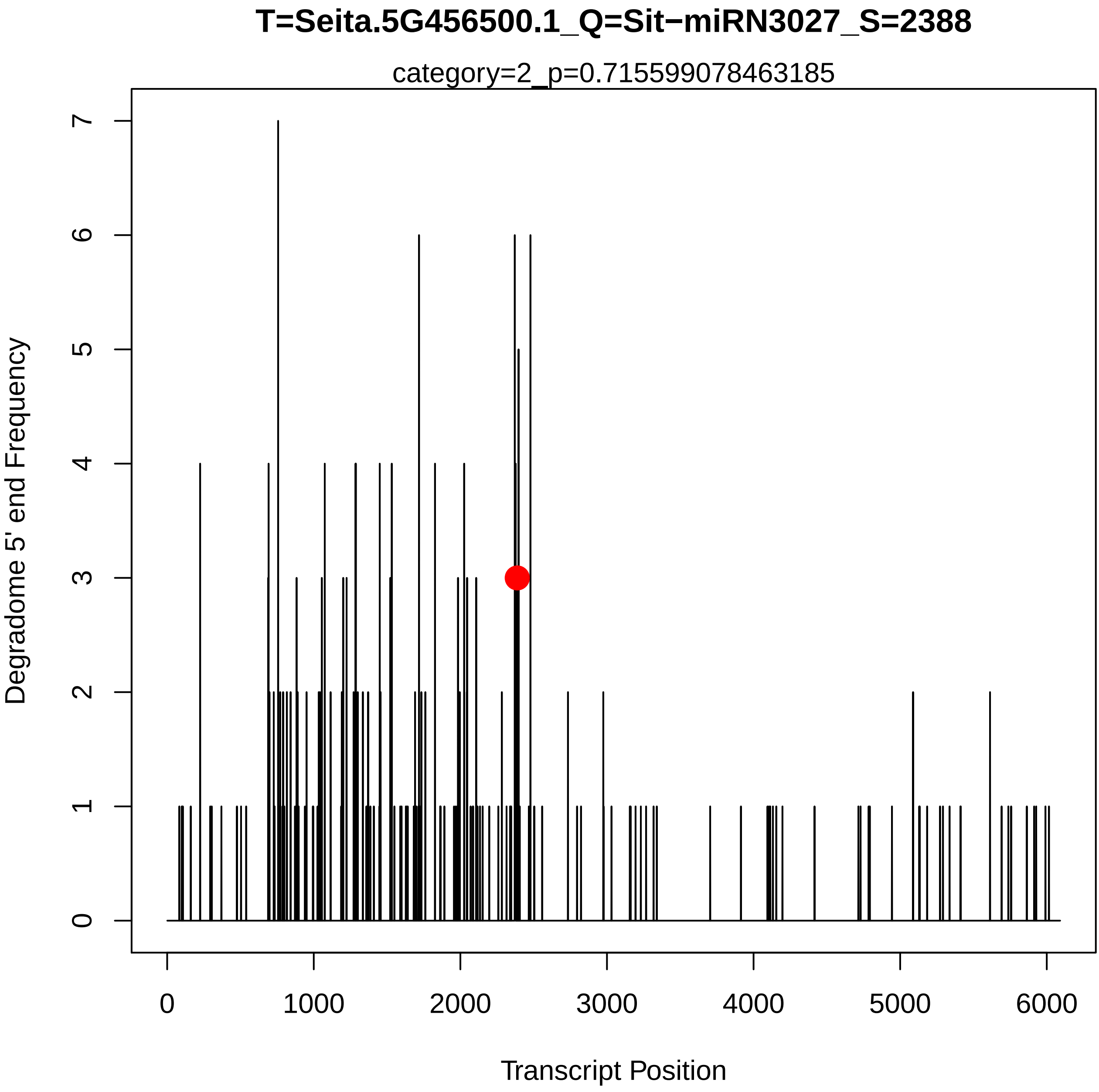
T=Seita.5G456500.1_Q=Sit−miRN3027_S=2388
category=2_p=0.715599078463185
Degradome 5' end Frequency
0
1
2
3
4
5
6
7
0
1000
2000
3000
4000
5000
6000
Transcript Position
